# Nutritional Determinants of Quality of Life in a Mediterranean Cohort: The SUN Study

**DOI:** 10.3390/ijerph17113897

**Published:** 2020-05-31

**Authors:** Octavio Pano, Carmen Sayón-Orea, Alfredo Gea, Maira Bes-Rastrollo, Miguel Ángel Martínez-González, J. Alfredo Martínez

**Affiliations:** 1 Department of Preventive Medicine and Public Health, University of Navarra, School of Medicine-Clínica Universidad de Navarra, 31008 Pamplona, Spain; opano@alumni.unav.es (O.P.); ageas@unav.es (A.G.); mbes@unav.es (M.B.-R.); mamartinez@unav.es (M.Á.M.-G.); 2 IdiSNA, Navarra Institute for Health Research, 31008 Pamplona, Spain; jalfmtz@unav.es; 3 Navarra Public Health Institute, 31003 Navarra, Spain; 4 Área de Fisiopatología de la Obesidad y la Nutrición (CIBEROBN), Centro de Investigación Biomédica en Red, 28049 Madrid, Spain; 5 Department of Nutrition, Harvard T.H. Chan School of Public Health, Boston, MA 02115, USA; 6 Department of Food Sciences and Physiology, University of Navarra, Center for Nutrition Research, 31008 Pamplona, Spain; 7 Precision Nutrition and Cardiometabolic Health Program, IMDEA Food Institute, 28049 Madrid, Spain

**Keywords:** health related quality of life, SF-36, Mediterranean, provegetarian, lifestyle

## Abstract

Health related quality of life (HRQoL) is a subjective appreciation of how personal characteristics and health influence well-being. This cross-sectional analysis aimed to quantitatively measure the influence of dietary, lifestyle, and demographic factors on HRQoL. A sub-sample of the Seguimiento Universidad de Navarra (SUN) Project, a Mediterranean cohort, was analyzed (n = 15,674). Through self-administered questionnaires the relationship between HRQoL and dietary patterns (Mediterranean-diet (MedDiet) and provegetarian food pattern (FP) assessment), lifestyles (sleeping hours, physical activity) and demographic characteristics were measured. Multivariate linear regression and flexible regression models were used to estimate the pondered effect of personal factors on Short Form-36 (SF-36) scores. Coefficients for MedDiet and provegetarian scores (β-coefficient for global SF-36 score: 0.32 (0.22, 0.42); 0.09 (0.06, 0.12) respectively for every unit increase), physical activity (β: 0.03 (0.02, 0.03) for every metabolic equivalent of task indexes (MET)-h/week) had a positive association to HRQoL. The female sex (β: −3.28 (−3.68, −2.89)), and pre-existing diseases (diabetes, β: −2.27 (−3.48, −1.06), hypertension β: −1.79 (−2.36, −1.22), hypercholesterolemia β: −1.04 (−1.48, −0.59)) account for lower SF-36 scores. Adherence to MedDiet or provegetarian FP, physical activity and sleep are associated with higher HRQoL, whereas the female sex, “other” (versus married status) and the presence of chronic diseases were associated with lower SF-36 scores in this sample.

## 1. Introduction

Quality of life (QoL) is a concept recognized by the World Health Organization (WHO) to describe an individual’s perception of their environment, socioeconomic status, personal and demographic characteristics [1,2]. Each of these factors, both internal and external, can influence individual and population health [3,4]. These factors are defined as determinants of health, all of which have varying degrees of impact on QoL as a result of personal standards, morals, and experiences in addition to ageing [4]. Given the interest for the impact and complex interactions of these determinants, the WHO and other scientific groups (Euro-QoL group, Nottingham University, the Research and Development (RAND) corporation, etc.) have attempted to measure how health status affects QoL [5,6,7]. Health related quality of life (HRQoL) are the set of determinants that influence how health is perceived by an individual [2,8]. Demographic characteristics, personal regard for healthcare-services, and the status of peers are just some of the concepts that influence HRQoL [1]. The term QoL holds great importance when measuring health as it is an acknowledgment that the well-being of a population or patient must guide clinical practice and public policies, both in the presence and absence of disease [2,9]. 

Traditionally, HRQoL has been assessed through self-administered questionnaires which have been adapted to different languages and social scenarios to account for cross-cultural differences [10,11,12]. In general terms, HRQoL is determined by factors that stem from physical, mental and social spheres [13]. The implementation of these questionnaires has given insight into the associations between health-experience and nutritional habits, frequency of medical visits, incidence of metabolic-diseases, predicting rehospitalization, some biomarkers, and even mortality [8,14,15,16,17]. One of the most commonly used questionnaires is the Short Form-36 (SF-36) (developed for the Medical Outcomes Study (MOS)), a tool that has gradually gained interest alongside HRQoL. The SF-36, as other HRQoL assessment tools, focuses on the impact of health on various aspects of an individuals’ experience, such as physical capabilities, psychological as well as emotional well-being [13]. The questionnaire developed for the MOS is only one of many others but has been appraised for its reliability and practicality for individual or population studies [18]. In this context, HRQoL has been associated to factors that range from personal characteristics (depressive symptoms, marital status) to a much broader sense, such as economic status of a region [15,18,19]. Interestingly, inverse associations between key markers of insulin resistance such as fasting plasma glucose, glycosylated hemoglobin and the homeostatic model assessment index for insulin resistance (HOMA-IR) in relation to HRQoL have been described [15,20]. In light of these findings, it may be of interest to explore the possible uses of HRQoL as an identifier of patients at risk due to a low perception of health. 

Two of the most representative determinants of HRQoL at an individual level, are dietary quality and physical activity. Studies show that these factors improve HRQoL but the inverse is also true, where a negative outlook of health can discourage from adhering to a healthy diet or performing physical activity [21,22]. Whilst previous research establishes the existence of a relationship between nutrition, lifestyle and HRQoL, quantifications of these effects have not been determined. Hence, the objective of this study was to quantify the relationships between nutritional, lifestyle, personal factors and HRQoL using the SF-36. The resulting measurements would correspond to the portion of HRQoL determined by the presence, absence or degree of each personal characteristic which could give greater insight into the impact of HRQoL on daily activities.

## 2. Materials and Methods

### 2.1. Study Population

The Seguimiento Universidad de Navarra (SUN) study is a prospective, and multipurpose cohort conducted in a Mediterranean setting in Spain. Enrollment in the study is permanently open under the identifier NCT02669602 at ClinicalTrials.gov and a detailed description of the methods is available elsewhere [23]. Briefly, this dynamic cohort started recruitment in December of 1999 with the goal of establishing associations between diet and the occurrence of several diseases and chronic conditions. The cohort consists of post-graduate students from universities across Spain, who are invited to participate in this study. Data collection and follow-up is done through biennial (mailed or web-based) questionnaires, gathering information on the various outcomes of interest. The questionnaires vary at each follow-up to assess various outcomes but are similar across all participants despite being enrolled at different time periods. At baseline, information on diet, lifestyle, risk factors, and medical conditions are collected and thereof various outcomes such as QOL, use of medications, recently diagnosed diseases, among others are investigated. 

Participants gave consent for their data to be stored and used to contact them for advances and current developments in the cohort to promote retention within the study. Potential candidates were also informed of their right to refuse to participate in the SUN study or to withdraw their consent to participate at any time without reprisal, according to the principles of the Declaration of Helsinki. The voluntary completion and submission of the baseline questionnaire was considered to imply informed consent. The Research Ethics Committee of the University of Navarra approved this method to request informed consent of participants and the entirety of the SUN-Cohort Study’s methods under the code 2001/30.

**Figure 1:**
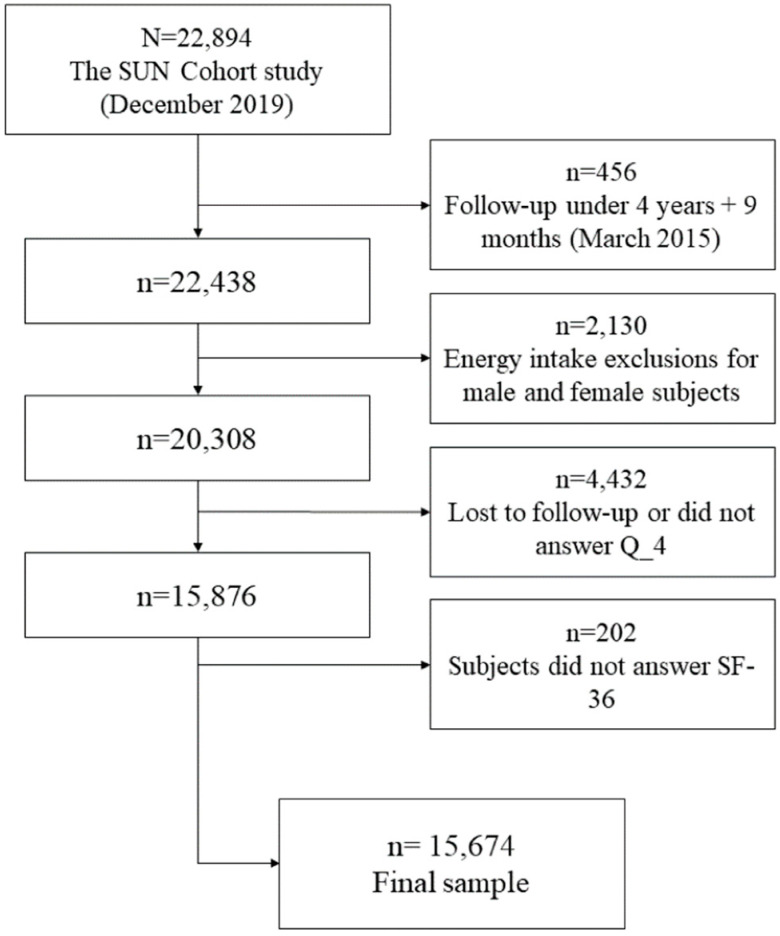
Flow chart and exclusion criteria for the present sample.

For this cross-sectional study, a subsample of *n* = 15,674 subjects were analyzed. The latest database of the SUN cohort study (from December 2019) contained data on 22,894 university graduates, some of which have been enrolled since 1999. From this sample, a subset was extracted based on the exclusion criteria on Figure 1. Exclusion criteria included a minimum follow-up of four years and nine months or those included in the study after March of 2015 despite the cross-sectional nature of this nested analysis. This cut-off was set to exclude participants who had not yet submitted information of the SF-36 questionnaire which is evaluated at the fourth year of follow-up (Q4) for each participant. The additional nine months was an estimate of time necessary for participants who had been in the study for at least four years to submit their questionnaire and for this data to be included in the database. A total of *n* = 456 participants had a follow-up under four years and nine months. Other exclusion criteria were: reports of implausible total energy intake for male (<800 kcal/day or >4000 kcal/day) and female subjects (<500 kcal/day or >3500 kcal/day) (*n* = 2130), lost to follow-up or did not answer Q_4 (*n* = 4432), and participants who did not answer the SF-36 questionnaire (*n* = 202). Despite the inclusion of a minimum follow-up period, all analyses were conducted in a cross-sectional form.

### 2.2. Dietary and Non-Dietary Variables

The information collected in the baseline questionnaire (Q_0) included participants’ dietary information (described below), medical history (prevalence of chronic diseases such as cancer, diabetes and cardiovascular disease), health related habits (smoking status), lifestyle (physical activity during leisure time, sleeping hours), and socio-demographic variables (sex, age, marital status), as well as anthropometric data (weight and height).

Among demographic variables, we studied marital status as one of five categories: single, married, widow, separated and for those who did not identify with any of these categories the item “other” was included. Personal history of diseases consisted of dichotomic variables for diabetes, hypertension and hypercholesterolemia when diagnosed by a trained physician and self-reported by the participants. Once participants report the diagnosis of diabetes, they are contacted to confirm the diagnosis and presented with a supplementary questionnaire to further investigate the initial report. For the particular case of hypertension, a validation study was conducted in which the authors concluded that the self-reported diagnosis on hypertension is reliable in our cohort [24]. Family history of diseases (FHD) included parental history of obesity, cardiovascular disease, infarction, hypertension, diabetes, and some forms of cancer (pulmonary, melanoma, colon and breast cancer). Smoking status was interrogated as a categorical variable according to the current smoking status at baseline of participants as one of three categories: never smoked, current smoker and formerly smoked. It was previously described that participants of this cohort report lower HRQoL in association to smoking status and dosage per day [25]. Body mass index (BMI) was calculated based on the self-reported data for weight (kg) and height (m), validity of the data is reported elsewhere [26]. In short, self-reported height and weight were interrogated and compared to measured data in order to verify their reliability to classify individuals. Furthermore, sensibility and specificity were assessed for overweight and obese individuals based on their self-reported data and authors conclude that this data is reliable for use in further analyses. To quantify leisure time physical activity (LTPA) participants were asked on the frequency with which they performed 17 activities in the past year: walking, jogging, stationary cycling, among others. For each of these activities metabolic equivalent of task indexes (MET) were estimated. Once frequencies are obtained, these were multiplied by the standard METs exerted while performing each activity, and then averaged to obtain the total METs per hour per week (METs-h/week). This methodology mimicked that of two American cohorts and was further validated in this cohort [27,28,29]. Sleeping hours were analyzed as night-time and nap-time sleep, based on the reported weekly and weekend habits. In case of night-time sleep, 12.3% of data was missing and through a multivariate regression method, imputed. The variables sex, age, BMI, LTPA, and time spent with friends were used as imputing variables for the multivariate regression model for the command “impute” of the statistical program STATA version 14.0 (StataCorp, 2015, Stata Statistical Software: Release 14. College Station, TX, USA: StataCorp LP). Further categorization of sleeping hours was done in three consecutive categories for those who slept below 7 h/day, 7–8 h/day, and above 8 h/day based on observations and recommendations from previous authors [30,31] and evidence on the link between obesity, diabetes, cardiovascular disease, as well as hypertension and sleeping hours [31,32]. Daily nap-time sleep was categorized as a dichotomic variable to differentiate between participants who slept above or below 30 min/day in the form of naps. This data was analyzed separately from total sleep time. The definition of this threshold was based on a previous study where nap-time sleep was observed to have protective properties in the development of obesity below the 30 min/day cut-off [33]. 

Information on dietary intake and habits was collected using a validated semi-quantitative food frequency questionnaire (FFQ) comprising 136 food items at Q_0 [34,35,36]. Participants were asked to report the frequency of consumption in standard Spanish servings (servings/day) of foods and food groups [37]. Further treatment of the variable consisted in transforming the consumption to total grams per day (g/day) of each food and food groups. Frequency of consumption was measured in nine categories ranging from never/almost never to >6 servings/day for each food item. 

Dietary quality was measured as adherence to a Mediterranean dietary pattern as defined by Trichopoulou et al. in 2003 [38,39]. The Mediterranean-diet scale (MedDiet) takes into consideration nine items [ratio of monounsaturated over saturated fats (MUFA/SFA), fruits and nuts, vegetables, cereals, legumes, fish, meat, dairy, and alcohol] regarded as characteristic of a Mediterranean diet pattern. Consumption above the median for the items MUFA/SFA, fruits and nuts, vegetables, cereals, legumes, and fish award one point per category. Whereas consuming below the median of the sample in the items meat and dairy products award one point in each category. Regarding alcohol consumption, moderate intake was considered beneficial and a habit that falls in line with a Mediterranean lifestyle. Prior to this study, a moderate consumption of alcohol in other studies also in a Mediterranean setting have found beneficial properties of a low intake of alcohol and the avoidance of binge drinking [25]. In the MedDiet scale, one point is awarded for male participants who consumed between 10–50 g/day and 5–25 g/day for female subjects. Scores are pooled and range from zero to nine, lowest to highest adherence to the dietary pattern, and further categorized into three groups of low (0–2 points), moderate (3–6 points), and high adherence (7–9 points) to this pattern. Diet was additionally analyzed using a provegetarian FP assessment score described by Martinez-Gonzalez, MA. et al. in the past and previously examined in this cohort [40,41]. This pattern takes into consideration energy-adjusted consumption of various food groups characteristic of a provegetarian FP and uses quintiles to assign points. Energy-adjustments were performed separately for each sex. For the food groups: fruits, vegetables, nuts, legumes, cereals, olive oil and potatoes, points were assigned in relation to the quintile to which they were allocated (quintile one received 1 point, quintile two received 2 points, and so on). On the other hand, the food groups: animal fats, eggs, fish, dairy products, meat and meat products quintile scoring was inverted (quintile one received 5 points, quintile two received 4 points, quintile three received 3 points, etc.). Scores were then pooled in a discontinuous scale from 12 points (lowest possible adherence to the FP) to 60 points (highest adherence to the FP). Additional categories of the scale were created for a total of four groups: very low (12–30 points), low (31–34 points), moderate (35–39 points), and high (40–60 points) adherence to the provegetarian FP. The authors described an additional “very high” adherence category, but due to a low sample in the group (*n* = 25), these subjects were pooled into the “high” adherence category [40]. 

### 2.3. Main Outcome—Health Related Quality of Life (HRQoL) the SF-36 Questionnaire

At Q_4 (four years of follow-up) the assessment of HRQoL was performed using a validated Spanish-language SF-36 Health Survey [12,42]. The survey contains 36 items measuring eight multi-item components of health status: (1) physical function, (2) role limitations due to physical health (role physical), (3) bodily pain and (4) general health, which corresponded to a physical domain (PhC); and (5) role limitations due to emotional problems, (6) energy/fatigue, (7) emotional well-being and (8) social functioning which correspond to a mental domain (MeC). As described by the authors, each parameter is scored, summed and transformed to a scale from 0 (the worst possible quality of life) to 100 (the best possible quality of life) [13]. Reports of overall health perception were done through three cumulative scores, summary scores, which assessed either physical or mental spheres (PSph and MSph) and a global HRQoL score which pondered every component in the questionnaire. Furthermore, the SF-36 includes an assessment of changes in perceived health over the past year through one item of the questionnaire. Subjects report changes on a five option Likert-style item ranging from “much better now than one year ago” to “much worse now than one year ago”.

### 2.4. Statistical Analysis

Statistical analyses were carried out using STATA version 14 (StataCorp, 2015, Stata Statistical Software: Release 14, College Station, TX, USA: StataCorp LP). Determinants of HRQoL were analyzed as continuous [age, sleeping hours (h/day), BMI, total energy intake, physical activity (METs-h/week) and provegetarian FP], categorical [marital status, sleeping hours (<7 h/day, 7–8 h/day and >8 h/day), BMI for sample characterization (based on the WHO’s definition for obesity), provegetarian FP and MedDiet], and dichotomous variables [sex, pre-existing diabetes, pre-existing hypertension, pre-existing hypercholesterolemia, family history of chronic diseases, and individual components of the MedDiet score]. Normality was assumed due to sample size and verified using graphical means. Comparisons of demographic, lifestyle and individual characteristics across categories of MedDiet score were performed using Students t-test of equal or unequal variances, or analysis of variance (ANOVA) for parametric continuous variables, in addition to the Dunnett method to define differences between groups. Statistically analysis for categorical variables were done using proportions analysis, using χ^2^ distribution. 

Accompanying analysis included the study of personal features and their distribution amongst participants with low or high HRQoL. Stratification in either low or high HRQoL was based on the median SF-36 global summary score (80.94 points) as the cut-off value. For the multivariate linear regression models a linear relationship between HRQoL (SF-36) and each continuous variable was verified. Furthermore, normality was studied using graphical means on the predicted residuals of each multivariate model. Given the sample size, reasonable adjustment to normality and robustness of linear regression models, this method of analysis was considered appropriate. Multivariate linear regression models were carried out for the eight components of the SF-36 and for the summary scores (global, PSph, MSph and transition scores) as dependent variables. Adjustment of the models was done for the afore mentioned lifestyle, demographic characteristics and the MedDiet score (except for the model containing the provegetarian FP) as independent variables in a continuous or categorical format in order to obtain β-coefficients and 95% confidence intervals (CI) for each factor. In the analysis of categorical variables, literature-based reference categories were used when possible (marital status, sleeping hours, and BMI), or the absence of the factor. β-coefficients reflect the amount of SF-36 points that are attributed to each individual factor. The provegetarian FP was categorically analyzed in independent regression models for each summary component score (dependent variable), and similarly adjusted for lifestyle, and demographic factors as the MedDiet models.

Sensitivity analysis was conducted using non-imputed data. Subgroup analyses by sex and pre-existing diabetes were also conducted.

Furthermore, to better understand the relationship between diet quality and HRQoL, flexible regression models (cubic splines) were used to obtain the tier-specific odds ratios (OR) for high global SF-36 score (outcome), using the MedDiet or the provegetarian FP as continuous associated variables. Each model was controlled for sex, age, BMI, marital status, pre-existing diabetes, pre-existing hypertension, pre-existing hypercholesterolemia, family history of diseases, total physical activity, total sleeping hours and total energy consumption.

## 3. Results

### 3.1. Sample Characteristics and Overall HRQoL Scores

After applying our exclusion criteria, the final sample consisted of 15,674 participants (Figure 1), primarily female (59.8%), with an overall mean age of 38.5 years (SD; 12.1) (Table 1). From the analyzed pre-existing diseases, hypercholesterolemia was the most prevalent (17.9%), mean BMI was 23.6 kg/m^2^ (3.5), and the average LTPA was 21.6 METs-h/week (22.6). Regarding dietary quality the mean MedDiet score was 4.2 points (1.8), and 30.0 points (5.0) for the provegetarian FP. A moderate correlation was found between MedDiet and provegetarian FP scores, Pearsons r: 0.60 (*p* value <0.001). Amongst categories of adherence to a Mediterranean dietary pattern it is noteworthy that 43.1% of men have a high adherence to the MedDiet pattern, the progressively higher mean age of participants from the low to high adherence categories [low: 34.5 points (10.3) and high adherence: 43.7 points (12.7)] and the higher energy intake for those with highest adherence to a Mediterranean dietary pattern; (2549 Kcal/day (543) vs. 2202 Kcal/day (593) in the low adherence group), *p* value <0.05 for ANOVA testing. 

Additional characteristics of the sample are shown in Table 2. Male participants were found to score higher than their female counterparts in the MSph (difference of 2.3 points) and global score (difference of 2.7 points), but the greatest differences were found in the PSph (difference of 3.1 points), *p* < 0.05. Individuals without chronic diseases scored consistently higher in the global and PSph SF-36 scores and were most notable for pre-existing diabetes in the PSph [80.3 points (0.8) versus 85.1 (0.1) for non-diabetic, *p* < 0.05].

### 3.2. Dietary Analysis

#### 3.2.1. Multivariate Linear Regression Models

Table 3 includes β-coefficients (CI) of sociodemographic and personal characteristics as the attributed influence on HRQoL. For global SF-36, the factors that were associated the lower scores were female sex [hereafter; β-coefficient −3.28 (95% CI: −3.68, −2.89), pre-existing diabetes −2.27 (−3.38, −1.06) and the category “other” for marital status −1.38 (−2.72, −0.02)]. Across summary scales, only sex was significantly associated with lower global SF-36 (*p* < 0.05), followed by age which was associated with a higher only in the MSph score (0.07 (0.05, 0.09)) but with lower scores in the remaining summary scores. Physical activity was linked to higher scores across all summary measures [for the global score 0.03 (0.02, 0.03)], and the opposite was observed for sleeping hours at night in both comparison categories [β-coefficient <7 h/day of sleep: −1.10 (−1.54, −0.67) and for >8 h/day of sleep: −0.68 (−1.22, −0.14)]. Sensitivity analysis performed on non-imputed data for sleeping hours (*N* = 13,750) was conducted, changes in results were not meaningfully (data not shown). Subgroup analysis by sex (female subjects *N* = 9369; male subjects *N* = 6305) on Tables S1–S2, revealed similar results for both groups across all summary scores and individual factors. Moreover, when analyzing possible interactions between pre-existing diseases, results revealed similar effects for both male and female subjects in the presence of pre-existing diabetes (*N* = 293) compared to their counterparts (*N* = 15,381) in the global, physical and mental scales, *p* values for interaction = 0.401, 0.278 and 0.812 respectively.

In general, dietary quality (MedDiet elements and provegetarian FP categories) (Table 4) was associated with higher HRQoL across summary component scores. Amongst dietary items the category of highest adherence to a provegetarian FP was attributed the highest improvements on HRQoL scores, 2.39 points (0.99, 3.80) of the transition scale. In relation to the MedDiet score, fruit consumption was related to a 0.80 point (0.41, 1.20) increase in the MSph, was the highest scoring item in the global score and had consistently significant results across all summary scores. Vegetable consumption was associated with higher scores for most of the summary scales, being highest for the transition scale, β-coefficient of 0.80 SF-36 points (0.28, 1.32) followed by the physical scale with 0.60 (0.15, 1.05). Among other findings, fish consumption and an adequate MUFA/SFA consumption were amounted for higher HRQoL scores in the MSph, 0.51 (0.14, 0.89) and 0.63 (0.25, 1.01) respectively. 

#### 3.2.2. Flexible Regression Models (Cubic Splines)

**Figure 2:**
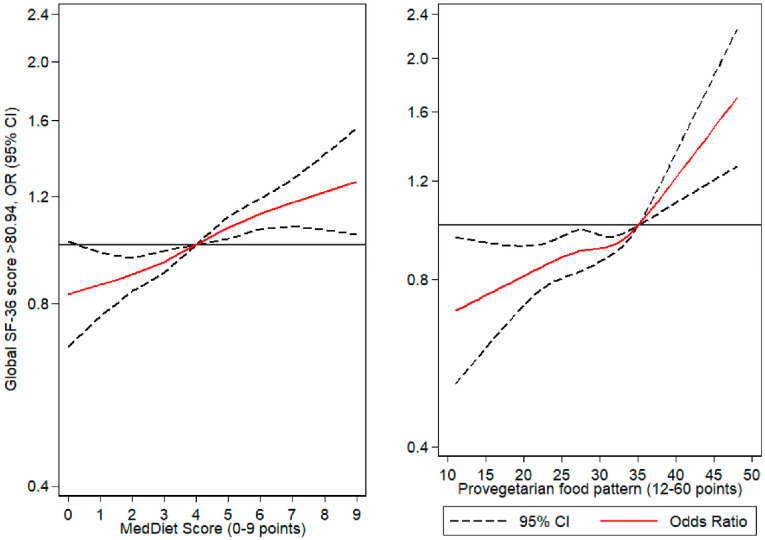
Flexible regression models, cubic spline analysis. Odds ratios (solid line) and 95% CI (dotted lines) for higher than the median global SF-36 score (80.94 pts.) in relation to the MedDiet and Provegetarian FP score. Reference category was set to the median of each dietary score.

In Figure 2, we found that scoring under four points of the MedDiet score the probability of having a high HRQoL decreased in comparison to four points. These observations were non-significant for those who scored 0 points. On the other hand, scores above four points increased the OR of having high global SF-36. For the provegetarian FP, all participants under the defined threshold (35 points) had reduced OR for high HRQoL. In line with our previous results, those who scored above this limit had an increased OR (nearly 60% increase) of scoring above the median in global HRQoL. Additional tables on the regression models for each domain of the SF-36 are presented in the Tables S3–S4. In short we observed varying degrees of association between the various SF-36 domains and the dietary and lifestyle characteristics. Most notably were the impacts of female sex on physical domains such as bodily pain (β: −6.45 (−7.16, −5.75)) and role physical (β: −5.88 (−6.87, −4.88)). Pre-existing diseases were associated with lower HRQoL across physical and mental domains, of which the highest reductions in HRQoL scores were found in subjects with diabetes and their scores for general health (β: −7.74 (−9.23, −5.85)).

## 4. Discussion

In our study we found that there is a positive association between HRQoL, as measured by the SF-36 and diet quality, both as a Mediterranean pattern or a provegetarian FP. These associations were also found for sleeping hours and LTPA, whereas female sex, age, marital status (other vs single), and pre-existing diseases (diabetes, hypertension and hypercholesterolemia) were associated with lower HRQoL scores. The measurement of HRQoL to assess an individual’s experience of health has been associated with clinical markers or used as a prognostic-indicator for various diseases in the past [43,44,45,46]. Previous research has highlighted the role of nutrition and various lifestyle factors on this health assessment [21,47,48,49]. In the SUN cohort, analysis of the impact of diet quality had been previously performed describing the positive impact of a high-quality diet on HRQoL [50]. Our study is in agreement with these observations but focuses on the associated SF-36 scores, as a valid and reliable assessment of health perception, to each unit/category of a health feature and not solely the adjusted HRQoL scores in each diet group [11,18].

Furthermore, the use of cubic splines allowed us to study relationships between MedDiet and a provegetarian FP in a non-linear fashion in addition to the individual probability of scoring above or below the median SF-36 score of our sample. This non-linear analysis allows us to describe the relationship between HRQoL and diet patterns, an association that should be studied in depth to identify causal relationships in a future study with a prospective format. Moreover, physical activity, BMI, sleeping hours, pre-existing diseases and marital status significantly affect HRQoL, speaking to the importance of overall healthy habits and their association to health perception.

Regarding our analyses, the estimation of β-coefficients in a conjunct model for each summary score allows for quantitative comparisons between each determinant of health, in varying degrees, and the individual impact of each factor on HRQoL. These relationships were previously explored in a pilot study of a small Spanish sample [51], using the SF-36 as a valid and reliable tool as previously demonstrated [12,42]. Our results confirm these results, but offer higher precision as to the impact of each feature on SF-36 scores. Prior to our study, the importance of daily habits and individual characteristics and their associations with HRQoL had been suggested [52,53]. Hence, each β-coefficient would represent the associated increase or decrease in SF-36 scores for each unit of the independent variable, or when the criteria for a given category is met (categorical variables). For the particular case of sex, we presented the β-coefficients (95% CI) for female subjects when compared to their male counterparts. Subgroup analyses by sex were conducted to identify sex specific β-coefficient and possible interactions, although none were found. Further analysis of interactions between coexisting characteristics should be researched in depth as the current study is limited to the description of these associations. 

When categorizing subjects according to the degree of adherence to a Mediterranean diet pattern we observed a similar distribution of participants in the provegetarian FP, reflecting the ability of both scores of identifying subjects with healthy eating habits. The category of highest adherence was characterized for performing more physical activity but also for the highest energy intake. These results have been described before in this cohort but are dissimilar to those in other Mediterranean samples where those with highest adherence to a Mediterranean FP had lower caloric intakes [23,54]. Despite not knowing the adequacy of the caloric intake of participants, the impact on health could be limited due to the high-quality diet and performing significantly higher physical activity as shown in our results.

For a better comprehension of HRQoL, minimum clinically significant changes (MCSC) were defined and are described by the authors of the MOS questionnaire [55]. These changes in SF-36 scores are set to define clinically relevant changes in scores (increase or decrease) which range from 2.5–5 points for any component or summary score. From the list of analyzed factors, only the female sex had such effects on global, PSph and MSph scores, highlighting the differences of how health is experienced between male and female participants. Among other factors that fulfill these MCSC are pre-existing diabetes and its association with PSph scores. In agreement with previous studies, the presence and degree of certain factors such as BMI and LTPA have varying effects on HRQoL [56,57]. The interpretation of our results and β-coefficients must be done in relation to the units in which each factor was analyzed, as continuous variables such as BMI reflect the SF-36 scores associated for each unit (kg/m^2^) of change, and categorical variables designate the associated β-coefficient when the criteria for a given category is met. With this notion, future studies could rely on the β-coefficients of modifiable factors as a mean to establish the necessary change in said variable, such as BMI, to yield MCSC.

In relation to dietary information and HRQoL, previous studies have noted the protective effect of a high-quality diet on self-perceived health [58]. Furthermore, weight-loss interventions can improve health perception, despite being outperformed by the combination of diet and exercise [48]. Our results on the Mediterranean-diet pattern are consistent with previous authors, which describe a direct association in PSph and MSph scores in relation to the adherence to this dietary pattern [54]. Furthermore, cubic spline analysis revealed that higher MedDiet adherence directly associate to higher ORs of scoring higher than the median SF-36 global scores. Interestingly fish or MUFA/SFA consumption, known for their neuroprotective qualities, predict an increase in MSph scores [59,60]. In relation to the non-significant results of meat consumption a possible explanation could be the categorization criteria, a method that was useful for other outcomes, but inconclusive for this particular fragment of the MedDiet score. Overall, similar results were observed in another Mediterranean-based cohort were higher adherence to a Mediterranean diet was associated with higher QoL as assessed by other means [61]. Our results not only confirm these findings but provide objective insight into the association of both dietary and non-dietary habits with HRQoL. 

When analyzing a provegetarian pattern we found similar results, were higher scores for this FP were associated with higher SF-36 scores, as seen in Table 1. Vegetarian diets and the particular case of the provegetarian FP have been associated with improvements in health for both overweight and obese individuals [41,62,63]. Prior research of the MedDiet and provegetarian food patterns as well as other dietary indexes note the elevated consumption of plant-based products with similar trends in cardiovascular disease prevention and overall metabolic health [62,63]. These associations of higher adherence to MedDiet and provegetarian FP were also noted in our study, finding a moderate correlation between scales as both patterns promote similar dietary habits. The use of cubic splines in the provegetarian pattern revealed that those with the highest adherence to this FP had higher HRQoL in comparison to those under 35 points, and to those who scored the highest in the MedDiet pattern. Possible explanations of these findings could stem from the health benefits conferred by a Mediterranean diet or provegetarian FP, further supported by specific reductions in SF-36 scores for components such as dairy products and meat consumption. Tentatively, the overall outlook of an individuals’ current health may influence dietary choices, hence a positive outlook could lead to healthier eating as evaluated by a Mediterranean or provegetarian pattern. This statement must be further researched in a prospective follow-up analysis in order to establish cause/effect relationships, and describe the interplay of habits, dietary choices and perceived health.

The main strengths of this study arise from the quantitative nature of our associations and the characteristics of the sample. Although the relationship between HRQoL and lifestyle factors had been previously studied, we defined a quantitative measure which describes the behavior of HRQoL in the context of each individual factor. The use of questionnaires is based on previously validated methods, providing solid information from which to draw conclusions. For the assessment of HRQoL, the SF-36 questionnaire has been widely used in various studies and has demonstrated consistency and adequacy in a sample of healthy participant. Further validation of the SF-36 has been performed comparing the results to disease-specific questionnaires, enabling us to draw conclusions for participants with the chronic diseases. In this study, our aim was to quantitatively assess the associations between lifestyle and dietary characteristics facilitating comparisons between factors with impact on HRQoL. The possible uses of our data could be applied to health surveys in the general population, or at an individual level. 

Due to the nature of our sample: young, university graduates of Spain the extrapolation of our results to other populations is limited. Still, the clear associations between HRQoL and healthy lifestyle and dietary quality are most likely to be found in the general adult population. One of the main draw backs of our study was the disparate measurement of diet and HRQoL as both variables are subject to change over time. In relation to this matter, our study aimed to describe associations, between daily habits and the associated health perception with disregard of the time at which the questionnaires were carried out. Furthermore, both the SF-36 questionnaire and the FFQ are valid tools of assessment over a period of time. On this basis, our study focused on dietary patterns in order to benefit from the habitual dietary practices of this Spanish, Mediterranean sample. In relation to the SF-36 questionnaire, regression to the mean has been noted over time referring to changes in scores by sex. For this reason, ageing and chronic diseases are objectively our main sources of HRQoL change. To this draw back, we would like to highlight the low prevalence of diseases in our sample and the apparently unchanged HRQoL scores reflected upon the scale “Transition” of the SF-36. To conclude it must be noted that substantial associations were described in our study and simultaneous measurements of HRQoL and lifestyle characteristics would only strengthen our findings. Further research should center on identifying cause-effect relationships. 

## 5. Conclusions

We conclude that a healthy diet and lifestyle (physical activity and sleeping hours) have a quantifiable, direct association with health perception. In contrast, there is an inverse association between pre-existing chronic diseases, unhealthy habits (sleeping more or less than 7–8 h/day, poor dietary quality and below the recommended daily physical activity) and demographic characteristics such as sex, age and marital status (category “other”) on the overall perceived health of an individual. These associations reveal that not only does each factor have a measurable influence on HRQoL but varying degrees of smoking status, sleeping hours, physical activity and diet quality have individualized associations.

## Figures and Tables

**Table 1:** Demographic and lifestyle characteristics of the total sample and as categories of the MedDiet score: The Seguimiento Universidad de Navarra (SUN) Study (2019).

Individual Characteristics	Total (*n* = 15,674)	MedDiet Score			*p*-Value
		Low (0–2 pts)	Moderate (3–6 pts)	High (7–9 pts)	
Sex (%, men)	40.2	39.4	40.0	43.1	*
Age (years)	38.5 (12.1)	34.5 (10.3)	38.8 (12.1)	43.7 (12.7)	*
Marital status: (%)					
Single	43.0	52.7	42.0	33.2	**
Married	52.2	44.5	52.9	60.0	
Widow	0.9	0.4	0.9	1.8	
Separated	2.5	1.5	2.6	3.1	
Other	1.5	0.9	1.6	1.9	
Diabetes (%)	1.9	0.8	2.0	2.8	**
Hypertension (%)	11.0	7.5	11.2	15.1	**
Hypercholesterolemia (%)	17.9	12.1	18.0	27.4	**
Family history of chronic disease (%)	63.8	58.7	64.2	69.8	**
Sleeping hours (h/day)	7.3 (0.8)	7.4 (0.8)	7.3 (0.8)	7.2 (0.8)	*
Siesta (min/day)	16.8 (43.2)	16.0 (44.9)	16.9 (43.0)	17.7 (41.6)	-
Body Mass Index (BMI) (kg/m^2^)	23.6 (3.5)	23.1 (3.5)	23.6 (3.5)	23.9 (3.6)	**
Total energy intake (kcal/day)	2344 (615)	2202 (593)	2349 (622)	2549 (543)	*
Mediterranean Diet Score (0–9 points)	4.2 (1.8)	1.6 (0.6)	4.4 (1.1)	7.3 (0.5)	*
Provegetarian Pattern (12–60 points)	30.0 (5.0)	25.7 (4.0)	30.3 (4.4)	35.4 (4.0)	*
Physical Activity (METs-h/week)	21.6 (22.6)	17.6 (20.2)	21.9 (22.4)	26.9 (25.8)	*

Values are presented as mean (SD) or proportions. Comparisons between groups were analyzed through ANOVA for normally distributed data, and proportions analysis using χ^2^ distribution for categorical variables. Abbreviations: METs: Metabolic equivalent of task. * = *p* value < 0.05, ** = *p* value < 0.001.

**Table 2:** Mean scores of the Short Form-36 (SF-36) global scale and subscales according to the baseline characteristics of participants.

Individual Characteristics	Global	Physical	Mental	Transition
Sex:				
Female	77.2 (0.1) *	83.7 (0.1) *	70.7 (0.1) *	50.9 (0.2) *
Male (ref.)	79.9 (0.1)	86.8 (0.2)	73.0 (0.1)	51.9 (0.2)
Age:				
<36 years (ref.)	78.9 (0.1)	86.6 (0.2)	71.1 (0.1)	52.6 (0.2)
>36 years	77.8 (0.1) *	83.4 (0.2) *	72.1 (0.1) *	50.1 (0.2) *
Marital status:				
Single (ref.)	78.1 (0.1)	84.6 (0.2)	71.5 (0.2)	51.5 (0.2)
Married	78.6 (0.1) *	85.4 (0.2) *	71.9 (0.1)	51.2 (0.2)
Widow	78.1 (0.9)	85.9 (1.2)	70.3 (1.0)	52.0 (1.3)
Separated	77.1 (0.5)	83.8 (0.7)	70.5 (0.6)	52.3 (0.8)
Other	76.6 (0.7) *	82.8 (0.9) *	70.3 (0.8)	52.9 (1.0)
Diabetes:				
No (ref.)	78.4 (0.1)	85.1 (0.1)	71.7 (0.1)	51.3 (0.1)
Yes	75.4 (0.6) *	80.3 (0.8) *	70.5 (0.7)	51.3 (0.9)
Hypertension:				
No (ref.)	78.6 (0.1)	85.4 (0.1)	71.8 (0.1)	51.3 (0.1)
Yes	76.4 (0.3) *	81.9 (0.3) *	70.2 (0.3) *	51.5 (0.4)
Hypercholesterolemia:				
No (ref.)	78.5 (0.1)	85.3 (0.1)	71.8 (0.1)	51.2 (0.1)
Yes	77.2 (0.2) *	83.5 (0.3) *	70.9 (0.2) *	52.0 (0.3) *
Family history of diseases				
None (ref.)	78.8 (0.1)	85.7 (0.2)	71.9 (0.2)	51.3 (0.2)
At least one disease	78.0 (0.1) *	84.5 (0.1) *	71.4 (0.1) *	51.3 (0.2)
Sleeping hours (h/day):				
<7	77.2 (0.2) *	84.2 (0.3) *	70.3 (0.2) *	50.8 (0.3) *
7–8 (ref.)	78.6 (0.1)	85.3 (0.1)	72.0 (0.1)	51.5 (0.1)
>8	77.7 (0.3) *	84.0 (0.3) *	71.4 (0.3)	51.2 (0.4)
Siesta (min/day)				
<30 (ref.)	78.5 (0.1)	85.3 (0.1)	71.8 (0.1)	51.3 (0.1)
>30	77.0 (0.2) *	83.2 (0.3) *	70.8 (0.2) *	51.3 (0.3)
BMI categories:				
Underweight	78.3 (0.4)	85.2 (0.6)	71.4 (0.5)	51.6 (0.6)
Normal (ref.)	78.7 (0.1)	85.6 (0.1)	71.8 (0.1)	51.2 (0.2)
Overweight	77.7 (0.2) *	84.0 (0.2) *	71.5 (0.2)	51.5 (0.3)
Obesity	75.6 (0.4) *	80.7 (0.5) *	70.5 (0.4) *	52.4 (0.6) *
Smoking Status:				
Never (ref.)	78.9 (0.1)	85.6 (0.2)	72.1 (0.1)	51.1 (0.2)
Current	77.4 (0.2) *	84.3 (0.2) *	70.5 (0.2) *	51.4 (0.3)
Former	78.0 (0.2) *	84.3 (0.2) *	71.7 (0.2)	51.6 (0.2)

Values are presented as mean (SD). * = *p* value < 0.05. Abbreviations: BMI, Body mass index. Prevalence of chronic diseases was self-reported by the participants. Family history of diseases refers to the parents of participants and includes data on pre-existing diabetes, hypertension, dyslipidemia, cardiovascular disease, and cancer. For dichotomic variables students T-test was performed for equal or unequal variances respectively. Polychotomous variables were compared using ANOVA, adjusted for age and sex, using Dunnet’s method to analyze differences against the reference group.

**Table 3:** Multivariable regression models with β-coefficients (95% CI) of lifestyle characteristics for each of the SF-36 questionnaire summary component scales.

Personal Characteristics	Global	Physical	Mental	Transition
Female (male ref.)	−3.28 (−3.68, −2.89) *	−4.06 (−4.57, −3.55) *	−2.50 (−2.94, −2.07) *	−0.84 (−1.43, −0.25) *
Age (years)	−0.03 (−0.05, −0.02) *	−0.14 (−0.16, −0.11) *	0.07 (0.05, 0.09) *	−0.16 (−0.19, −0.13) *
Marital Status				
Single (ref.)	0 (Ref)	0 (Ref)	0 (Ref)	0 (Ref)
Married	0.70 (0.29, 1.10) *	0.95 (0.43, 1.48) *	0.44 (−0.01, 0.89)	−0.44 (−1.04, 0.17)
Widow	0.32 (−1.44, 2.09)	1.63 (−0.68, 3.93)	−0.98 (−2.94, 0.98)	0.42 (−2.23, 3.07)
Separated	−0.74 (−1.83, 0.35)	−0.66 (−2.08, 0.76)	−0.82 (−2.03, 0.39)	0.63 (−1.00, 2.26)
Other	−1.38 (−2.72, −0.02) *	−1.57 (−3.33, 0.19)	−1.16 (−2.66, 0.33)	1.08 (−0.93, 3.09)
Pre-existing Diabetes	−2.27 (−3.48, −1.06) *	−3.75 (−5.33, −2.16) *	−0.79 (−2.14, 0.56)	−0.48 (−2.30, 1.33)
Pre-existing Hypertension	−1.79 (−2.36, −1.22) *	−2.39 (−3.13, −1.65) *	−1.20 (−1.83, 0.57)	0.06 (−0.79, 0.90)
Pre-existing Hypercholesterolemia	−1.04 (−1.48, −0.59) *	−1.37 (−1.95, –0.79) *	−0.70 (−1.20, −0.21) *	0.70 (−0.03, 1.36)
Family history of diseases Prev.	−0.65 (−1.00, −0.30) *	−0.91 (−1.36, −0.46) *	−0.40 (−0.79, −0.02) *	−0.15 (−0.66, 0.37)
Sleeping hours at night (h/day)				
<7	−1.10 (−1.54, −0.67) *	−0.68 (−1.25, −0.11) *	−1.53 (−2.01, −1.05) *	−0.77 (−1.42, −0.12) *
7–8	0 (Ref.)	0 (Ref.)	0 (Ref.)	0 (Ref.)
>8	−0.68 (−1.22, −0.14) *	−0.95 (−1.66, −0.25) *	−0.40 (−1.00, 0.20)	−0.16 (−0.96, 0.65)
Siesta (min/day)				
<30	0 (Ref.)	0 (Ref.)	0 (Ref.)	0 (Ref.)
>30	−1.09 (−1.55, −0.64) *	−1.57 (−2.16, −0.97) *	−0.62 (−1.13, −0.11) *	0.04 (−0.63, 0.72)
BMI (kg/m^2^)	−0.18 (−0.24, −0.12) *	−0.30 (−0.37, −0.23) *	−0.06 (−0.12, 0.003)	0.09 (−0.01, 0.18)
Physical activity (METs-h/wk)	0.03 (0.02, 0.03) *	0.03 (0.02, 0.04) *	0.03 (0.02, 0.03) *	0.02 (0.01, 0.03) *
Smoking status				
Never	0 (Ref.)	0 (Ref.)	0 (Ref.)	0 (Ref.)
Current	−1.26 (−1.68, −0.83) *	−1.14 (−1.69, −0.58) *	−1.38 (−1.85, −0.91) *	0.34 (−0.29, 1.00)
Former	−0.81 (−1.21, −0.41) *	−1.23 (−1.75, −0.71) *	−0.39 (−0.84, 0.05)	0.32 (−0.27, 0.91)

Each β-coefficient translates to the expected SF-36 points gained or lost per unit of age, total sleeping hours, BMI and LTPA or when the criteria of each individual factor is fulfilled. Individual regression models were performed for each of the summary scales, these are adjusted for the variables in this table in addition to elements of the Mediterranean diet score and total energy (kcal/day). Abbreviations: BMI, Body mass index; METs, Metabolic equivalent of task. Prev, prevalence of the chronic disease. If no reference category is specified, it was therefore set to the absence of the condition. Statistically significant results (*p* < 0.05) are presented with “*” on the table.

**Table 4:** Multivariable regression models with β-coefficients (95% CI) of dietary characteristics for each of the SF-36 questionnaire summary components.

Diet	Global	Physical	Mental	Transition
MedDiet	0.32 (0.22, 0.42) *	0.37 (0.25, 0.50) *	0.27 (0.16, 0.37) *	0.39 (0.25, 0.54) *
MUFA/SFA	0.45 (0.11, 0.80) *	0.28 (−0.17, 0.73)	0.63 (0.25, 1.01) *	0.11 (−0.41, 0.62)
Alcohol	0.53 (0.16, 0.89) *	0.81 (0.33, 1.29) *	0.24 (−0.17, 0.65)	−0.37 (−0.92, 0.18)
Cereal	0.16 (−0.20, 0.52)	0.21 (−0.26, 0.67)	0.11 (−0.28, 0.51)	−0.19 (−0.34, 0.72)
Vegetables	0.35 (−0.003, 0.69)	0.60 (0.15, 1.05) *	0.09 (−0.30, 0.48)	0.80 (0.28, 1.32) *
Fruits	0.74 (0.39, 1.09) *	0.68 (0.23, 1.14) *	0.80 (0.41, 1.20) *	0.81 (0.28, 1.33) *
Fish	0.39 (0.05, 0.73) *	0.27 (−0.18, 0.71)	0.51 (0.14, 0.89) *	0.31 (−0.20, 0.82)
Legumes	0.40 (0.06, 0.73) *	0.38 (−0.06, 0.82)	0.41 (0.04, 0.79) *	0.50 (−0.001, 1.01)
Dairy	−0.12 (−0.48, 0.24)	0.18 (−0.29, 0.65)	−0.42 (−0.82, 0.02)	0.44 (−0.10, 0.98)
Meat	−0.05 (−0.41, 0.30)	−0.01 (−0.46, 0.47)	−0.11 (−0.51, 0.28)	0.37 (−0.16, 0.90)
Provegetarian FP	0.09 (0.06, 0.12) *	0.11 (0.07, 0.15) *	0.07 (0.03, 0.11) *	0.14 (0.09, 0.19) *
Very Low	0 (Ref.)	0 (Ref.)	0 (Ref.)	0 (Ref.)
Low	0.59 (0.21, 0.98) *	0.66 (0.16, 1.16) *	0.53 (0.11, 0.96) *	0.40 (−0.17, 0.97)
Moderate	0.72 (0.24, 1.20) *	1.00 (0.38, 1.62) *	0.44 (−0.09, 0.97)	1.36 (0.65, 2.07) *
High	1.38 (0.44, 2.31) *	1.20 (−0.03, 2.42)	1.55 (0.51, 2.60) *	2.39 (0.99, 3.80) *

Each β-coefficient translates to the associated SF-36 points when the criteria of each individual factor is fulfilled when analyzing diet as either a Mediterranean or provegetarian FP. The Mediterranean dietary pattern was based on the score developed by A. Trichopoulou, in 2003 [38]. Individual multivariate linear regression models were performed for each of the summary scales, these are adjusted for the variables on Table 3 in addition to total energy intake. The models contain one of the two dietary quality assessment methods (MedDiet or provegetarian). Abbreviations: MUFA, monounsaturated fatty acids. SFA, saturated fatty acids. “Serv”, servings. Statistically significant (*p* < 0.05) results are presented with “*” on the table.

## Supplementary Materials

The following are available online at: https://www.mdpi.com/1660-4601/17/11/3897/s1, Table S1: Regression Models with β-regression coefficients and 95% confidence interval of the contributing dietary characteristics of the sample for the global and physical summary scores of the SF-36. Subgroup analysis by sex, Table S2: Regression Models with β-regression coefficients and 95% confidence interval of the contributing dietary and lifestyle characteristics of the sample for the mental and transition summary scores of the SF-36. Subgroup analysis by sex, Table S3: regression models with β-regression coefficients and 95% confidence for each of the physical and mental component scales, Table S4: regression models with β-regression coefficients and 95% confidence interval of the contributing dietary characteristics of the sample for each of the physical and mental subscale items.
