# Supplementary material for: Nutritional Determinants of Quality of Life in a Mediterranean Cohort: The SUN Study
Source: Int J Environ Res Public Health. 2020 May 31;17(11):3897. doi: 10.3390/ijerph17113897 (PMC7312060; doi:10.3390/ijerph17113897)
Supplement: Supplementary file 1 [file ijerph-17-03897-s001.pdf]

Table S1. Regression Models with β-regression coefficients and 95% confidence interval of the contributing dietary characteristics of the sample for the global and physical summary scores of the SF-36. Subgroup analysis by sex.

Personal Characteristics	Global		Physical	
	**Female (*N* = 9369)**	**Male (*N* = 6305)**	**Female (*N* = 9369)**	**Male (*N* = 6305)**
Age (years)	−0.02 (−0.49, 0.01)	−0.05 (−0.07, −0.02) **	−0.12 (−0.16, −0.08) **	−0.15 (−0.18, −0.11) **
Marital Status				
Single (ref.)	0 (Ref.)	0 (Ref.)	0 (Ref.)	0 (Ref.)
Married	0.14 (−0.39, 0.67)	1.47 (0.85, 2.10) **	0.41 (−0.29, 1.11)	1.67 (0.86, 2.47) **
Widow	−0.31 (−2.66, 2.03)	1.82 (−0.84, 4.48)	0.72 (−2.37, 3.80)	3.56 (0.13, 6.99) *
Separated	−0.29 (−1.70, 1.13)	−1.64 (−3.37, 0.09)	0.01 (−1.84, 1.87)	−2.07 (−4.29, 0.16)
Other	−1.56 (−3.34, 0.22)	−0.69 (−2.73, 1.34)	−1.74 (−4.08, 0.60)	−1.01 (−3.63, 1.62)
Pre-existing Diabetes	−2.49 (−4.58, −0.40) *	−2.26 (−3.68, −0.85) *	−4.38 (−7.13, −1.63) *	−3.49 (−5.32, −1.67) **
Pre-existing Hypertension	−2.48 (−3.47, −1.50) **	−1.61 (−2.27, −0.94) **	−3.27 (−4.57, −1.97) **	−2.11 (−2.97, −1.26) **
Pre-existing Hypercholesterolemia	−1.54 (−2.21, −0.88) **	−0.77 (−1.34, −0.19) *	−2.00 (−2.87, −1.12) **	−0.95 (−1.69, −0.21)
Family history of diseases Prev.	−0.92 (−1.39, −0.45) **	−0.17 (−0.67, 0.33)	−1.12 (−1.74, −0.50) **	−0.50 (−1.15, 0.14)
Sleeping hours at night (h/day)				
<7	−1.34 (−1.96, −0.73) **	−0.87 (−1.47, −0.26) *	−0.90 (−1.71, −0.10) *	−0.47 (−1.25, 0.31)
7–8	0 (Ref.)	0 (Ref.)	0 (Ref.)	0 (Ref.)
>8	−0.74 (−1.40, −0.09) *	−0.67 (−1.64, 0.31)	−1.00 (−1.87, −0.14) *	−1.04 (−2.30, 0.22)
Siesta (min/day)				
<30	0 (Ref.)	0 (Ref.)	0 (Ref.)	0 (Ref.)
>30	−0.89 (−1.52, −0.26) **	−1.49 (−2.13, −0.85) **	−1.40 (−2.22, −0.57) *	−1.91 (−2.74, −1.08) **
BMI (kg/m^2)	−0.25 (−0.32, −0.17) **	−0.07 (−0.16, 0.01)	−0.37 (−0.47, −0.27) **	−0.18 (−0.28, −0.07) *
Physical activity (METs-h/wk)	0.03 (0.02, 0.05) **	0.03 (0.02, 0.03) **	0.04 (0.02, 0.05) **	0.02 (0.01, 0.03) **
Smoking status				
Never	0 (Ref.)	0 (Ref.)	0 (Ref.)	0 (Ref.)
Current	−1.53 (−2.09, −0.97) **	−0.78 (−1.42, −0.15) *	−1.15 (−1.89, −0.42) *	−0.90 (−1.72, −0.09) *
Former	−0.91 (−1.46, −0.37) **	−0.63 (−1.20, −0.06) *	−1.27 (−1.99, −0.55) *	−1.07 (−1.80, −0.34) *
MedDiet (0/9 pts)	0.27 (0.13, 0.40) **	0.36 (0.22, 0.50) **	0.31 (0.14, 0.48) **	0.42 (0.24, 0.60) **

Each β−coefficient translates to the associated SF-36 points gained or lost per unit of age, total sleeping hours, BMI and LTPA or when the criteria of each individual factor is fulfilled. Separate multivariate regression models were performed for the global and physical scales of the SF-36 questionnaire and the listed personal characteristics as independent variables. The sample was categorized by sex and adjusted for total energy intake (kcal/day). Abbreviations: BMI, Body mass index; METs, Metabolic equivalent of task. Prev, prevalence of the chronic disease. If no reference category is specified, it was therefore set to the absence of the condition. Statistically significant results ($p < 0.05$) are presented with "*" or "**" for p values < 0.001 on the table.

Table S2. Regression Models with β-regression coefficients and 95% confidence interval of the contributing dietary and lifestyle characteristics of the sample for the mental and transition summary scores of the SF-36. Subgroup analysis by sex.

Personal Characteristics	Mental		Transition	
	Female (N = 9369)	Male (N = 6305)	Female (N = 9369)	Male (N = 6305)
Age (years)	0.08 (0.05, 0.11) **	0.06 (0.03, 0.08) **	−0.17 (−0.21, −0.13) **	−0.15 (−0.19, −0.11) **
Marital Status				
Single (ref.)	0 (Ref.)	0 (Ref.)	0 (Ref.)	0 (Ref.)
Married	−0.12 (−0.72, 0.47)	1.28 (0.59, 1.97) **	−0.41 (−1.17, 0.35)	−0.47 (−1.47, 0.53)
Widow	−1.34 (−3.96, 1.27)	0.08 (−2.87, 3.02)	1.30 (−2.09, 4.70)	−0.72 (−4.98, 3.54)
Separated	−0.58 (−2.16, 0.99)	−1.21 (−3.12, 0.70)	1.62 (−0.41, 3.64)	−1.02 (−3.78, 1.75)
Other	−1.38 (−3.37, 0.61)	−0.38 (−2.64, 1.88)	2.92 (0.36, 5.47) *	−1.88 (−5.17, 1.41)
Diabetes Prev.	−0.60 (−2.93, 1.73)	−1.03 (−2.60, 0.53)	−0.80 (−3.80, 2.20)	−0.23 (−2.50, 2.05)
Hypertension Prev.	−1.70 (−2.80, −0.60) *	−1.10 (−1.84, −0.37) *	0.42 (−1.00, 1.83)	−0.20 (−1.27, 0.86)
Hypercholesterolemia Prev.	−1.09 (−1.84, −0.35)*	−0.58 (−1.22, 0.05)	0.93 (−0.03, 1.89)	0.30 (−0.62, 1.23)
Family history of diseases Prev.	−0.72 (−1.24, −0.20) *	0.17 (−0.39, 0.71)	−0.23 (−0.91, 0.44)	0.01 (−0.80, 0.81)
Sleeping hours at night (h/day)				
<7	−1.79 (−2.47, −1.10) **	−1.26 (−1.93, −0.60) **	−1.19 (−2.07, −0.32) *	−0.22 (−1.18, 0.75)
7–8	0 (Ref.)	0 (Ref.)	0 (Ref.)	0 (Ref.)
>8	−0.49 (−1.22, 0.25)	−0.29 (−1.37, 0.79)	−0.27 (−1.21, 0.68)	−0.01 (−1.58, 1.56)
Siesta (min/day)				
<30	0 (Ref.)	0 (Ref.)	0 (Ref.)	0 (Ref.)
>30	−0.38 (−1.08, 0.32)	−1.07 (−1.78, −0.36) *	0.07 (−0.83, 0.97)	−0.09 (−1.12, 0.94)
BMI (kg/m^2)	−0.12 (−0.21, −0.04) *	0.03 (−0.06, 0.12)	0.11 (−0.01, 0.22)	0.09 (−0.04, 0.22)
Physical activity (METs−h/wk)	0.03 (0.02, 0.05) **	0.03 (0.02, 0.04) **	0.03 (0.01, 0.04) *	0.01 (−0.01, 0.03)
Smoking status				
Never (ref.)	0 (Ref.)	0 (Ref.)	0 (Ref.)	0 (Ref.)
Current	−1.91 (−2.53, −1.29) **	−0.66 (−1.36, 0.05)	0.06 (−0.74, 0.87)	0.86 (−0.16, 1.88)
Former	−0.56 (−1.16, 0.05)	−0.20 (−0.83, 0.43)	0.02 (−0.77, 0.80)	0.76 (−0.15, 1.68)
MedDiet (0/9 pts)	0.22 (0.08, 0.37) *	0.30 (0.14, 0.45) **	0.33 (0.14, 0.52) *	0.47 (0.24, 0.70) **

Each β-coefficient translates to the associated SF-36 points gained or lost per unit of age, total sleeping hours, BMI and LTPA or when the criteria of each individual factor is fulfilled. Separate multivariate regression models were performed for the mental and transition scales of the SF-36 questionnaire and the listed personal characteristics as independent variables. The sample was categorized by sex and adjusted for total energy intake (kcal/day). Abbreviations: BMI, Body mass index; METs, Metabolic equivalent of task. Prev, prevalence of the chronic disease. If no reference category is specified, it was therefore set to the absence of the condition. Statistically significant results ($p < 0.05$) are presented with "*" or "**" for p values < 0.001 on the table.

Table S3. Regression Models with β-regression coefficients and 95% confidence for each of the physical and mental component scales.

**Physical Components.**	**Physical Function**	**Role-Physical**	**Bodily Pain**	**General Health**
Sex	−3.01 (−3.37, −2.66) *	−5.88 (−6.87, −4.88) *	−6.45 (−7.16, −5.75) *	−1.09 (−1.71, −0.48) *
Age	−0.27 (−0.28, −0.25) *	−0.08 (−0.13, −0.03) *	−0.01 (−0.04, 0.03)	−0.19 (−0.22, −0.16) *
Marital Status[a]				
Single	0 (Ref.)	0 (Ref.)	0 (Ref.)	0 (Ref.)
Married	1.48 (1.11, 1.84) *	1.11 (0.10, 2.14) *	−0.12 (−0.84, 0.61)	1.42 (0.79, 2.04) *
Widow	0.29 (−1.30, 1.88)	4.72 (0.26, 9.17) *	−0.55 (−3.72, 2.62)	1.96 (−0.79, 4.70)
Separated	0.74 (−0.24, 1.72)	−2.25 (−5.00, 0.50)	−2.73 (−4.68, −0.77) *	1.59 (−0.11, 3.28)
Other	−0.34 (−1.56, 0.87)	−2.80 (−6.20, 0.60)	−1.37 (−3.79, 1.05)	−1.67 (−3.77, 0.42)
Pre-existing Diabetes	−2.96 (−4.06, −1.87) *	−3.94 (−7.00, −0.88) *	−0.53 (−2.71, 1.65)	−7.74 (−9.23, −5.85) *
Pre-existing Hypertension	−2.17 (−2.68, −1.66) *	−1.84 (−3.27, −0.41) *	−2.11 (−3.13, −1.09) *	−3.53 (−4.41, −2.65) *
Pre-existing Hypercholesterolemia	−0.44 (−0.84, −0.04) *	−1.60 (−2.72, −0.47) *	−1.48 (−2.28, −0.68) *	−1.94 (−2.63−1.25) *
Family history of diseases	0.05 (−0.26, −1.66) *	−0.99 (−1.86, −0.12) *	−1.43 (−2.05, −0.81) *	−1.21 (−1.75, −0.67) *
Sleeping hours (h/day)	−0.09 (−0.27, 0.09)	−0.04 (−0.54, 0.46)	0.37 (0.02, 0.73) *	0.27 (−0.04, 0.58)
Siesta (min/day)				
<30	1.01 (0.60, 1.42) *	2.34 (1.20, 3.48) *	1.51 (0.70, 2.32) *	1.84 (1.14, 2.54) *
>30	0 (Ref.)	0 (Ref.)	0 (Ref.)	0 (Ref.)
BMI	−0.36 (−0.41, −0.31) *	−0.24 (−0.38, −0.10) *	−0.39 (−0.49, −0.29) *	−0.21 (−0.30, −0.13) *
Physical activity (METs/w)	0.34 (0.03, 0.04) *	0.01 (−0.01, 0.03)	0.02 (0.01, 0.03)	0.05 (0.04, 0.06) *
Smoking Status				
Never	0 (Ref.)	0 (Ref.)	0 (Ref.)	0 (Ref.)
Current	−1.04 (−1.42, −0.65) *	−0.63 (−1.71, 0.44)	−1.34 (−2.10, −0.57) *	−1.61 (−2.27, −0.95) *
Former	−0.52 (−0.88, −0.16) *	−1.74 (−2.76, −0.73) *	−1.74 (−2.46, −1.02) *	−0.97 (−1.60, −0.35) *
**Mental Components**	**Role Emotional**	**Energy/Fatigue**	**Emotional Well-Being**	**Social Functioning**
Sex	−4.67 (−5.75, −3.58) *	−4.08 (−4.69, −3.47) *	−2.66 (−3.20, −2.13) *	1.17 (0.48, 1.85) *
Age	0.09 (0.04, 0.14) *	0.12 (0.09, 0.15) *	0.10 (0.08, 0.13) *	−0.04 (−0.07, −0.01) *
Marital Status				
Single	0 (Ref.)	0 (Ref.)	0 (Ref.)	0 (Ref.)
Married	2.47 (1.36, 3.58) *	0.44 (−0.19, 1.07)	−0.15 (−0.70, 0.40)	−0.85 (−1.55, −0.14) *
Widow	1.82 (−3.03, 6.68)	−0.63 (−3.37, 2.12)	−2.69 (−5.09, −0.29) *	−2.54 (−5.63, 0.55)
Separated	−0.48 (−3.48, 2.52)	−0.45 (−2.15, 1.24)	−0.94 (−2.43, 0.54)	−1.27 (−3.18, 0.64)
Other	−2.11 (−5.82, 1.60)	−0.35 (−2.44, 1.75)	−1.77 (−3.60, −0.06) *	−0.28 (−2.64, 2.08)
Pre-existing Diabetes	1.09 (−2.25, 4.43)	−2.53 (−4.42, −0.64) *	−0.48 (−2.13, 1.17)	−1.60 (−3.73, 0.53)
Pre-existing Hypertension	−1.92 (−3.48, −0.36) *	−2.04 (−2.92, −1.16) *	−1.21 (−1.98, −0.44) *	0.27 (−0.72, 1.27)
Pre-existing Hypercholesterolemia	−1.87 (−3.09, −0.64) *	−1.09 (−1.78, −0.40) *	−0.75 (−1.36, −0.15) *	0.95 (0.17, 1.73) *
Family History of diseases	−0.25 (−1.20, 0.70)	−0.96 (−1.49, −0.42) *	−0.35 (−0.81, 0.12)	−0.04 (−0.65, 0.56)
Sleeping hours (h/day)	0.96 (0.41, 1.50) *	0.44 (0.13, 0.74) *	0.94 (0.67, 1.21) *	0.01 (−0.34, 0.35)
Siesta (min/day)				
<30	1.03 (−0.21, 2.27)	1.37 (0.67, 2.07) *	0.87 (0.26, 1.48) *	−0.31 (−1.10, 0.48)
>30	0 (Ref.)	0 (Ref.)	0 (Ref.)	0 (Ref.)
BMI	−0.20 (−0.35, 0.05)	−0.11 (−0.20, −0.02) *	0.03 (−0.05, 0.10)	0.30 (−0.07, 0.13)

Physical activity (METs/wk)	0.04 (0.02, 0.06) *	0.06 (0.05, 0.08) *	0.04 (0.03, 0.05) *	−0.01 (−0.03, −0.01) *
Smoking Status				
Never	0 (Ref.)	0 (Ref.)	0 (Ref.)	0 (Ref.)
Current	−3.62 (−4.79, −2.45) *	−0.74 (−1.40, −0.08) *	−1.78 (−2.36, −1.20) *	0.58 (−0.16, 1.33)
Former	−0.76 (−1.86, −0.35) *	−0.45 (−1.07, 0.17)	−0.70 (−1.24, −0.15) *	0.27 (−0.43, 0.97)

Each β-coefficient translates to the expected SF-36 points gained or lost per unit of age, total sleeping hours, BMI and LTPA or when the criteria of each individual factor is fulfilled. Individual regression models were performed for each of the summary scales, these are adjusted for the variables in this table in addition to each item of the Mediterranean ,diet score and total energy (kcal/day). Abbreviations: BMI, Body mass index; METs, Metabolic equivalent of task. Prev, prevalence of the chronic disease. If no reference category is specified, it was therefore set to the absence of the condition. Statistically significant results ($p < 0.05$) are presented with "*" on the table.

Table S4. Regression Models with β-regression coefficients and 95% confidence interval of the contributing dietary characteristics of the sample for each of the physical and mental subscale items.

**Physical Components**	**Physical Function**	**Role-Physical**	**Bodily Pain**	**General Health**
Ratio MUFA/SFA	0.12 (−0.19, 0.42)	0.30 (−0.57, 1.17)	0.04 (−0.58, 0.66)	0.63 (0.09, 1.16) *
Alcohol	0.73 (0.40, 1.06) *	1.12 (0.19, 2.05) *	0.59 (−0.07, 1.25)	0.84 (0.27, 1.41) *
Cereal	0.30 (−0.02, 0.62)	−0.04 (−0.94, 0.86)	0.17 (−0.47, 0.81)	0.41 (−0.14, 0.96)
Vegetables	0.26 (−0.06, 0.57)	0.58 (−0.30, 1.46)	0.60 (−0.24, 1.23)	0.99 (0.44, 1.53) *
Fruits	0.80 (0.48, 1.11) *	0.19 (−0.70, 1.09)	0.50 (−0.13, 1.13)	1.27 (0.72, 1.82) *
Fish	0.30 (−0.01, 0.61)	0.40 (−0.46, 1.26)	0.23 (−0.38, 0.84)	0.16 (−0.37, 0.68)
Legumes	0.03 (−0.27, 0.33)	0.65 (−0.20, 1.50)	0.62 (0.02, 1.22) *	0.16 (−0.36, 0.68)
Dairy	0.13 (−0.19, 0.46)	0.41 (−0.50, 1.33)	0.10 (−0.55, 0.75)	0.07 (−0.49, 0.64)
Meat	0.15 (−0.17, 0.47)	−0.19 (−1.08, 0.71)	0.01 (−0.63, 0.65)	0.05 (−0.50, 0.60)
**Mental Components**	**Role-Emotional**	**Energy/Fatigue**	**Emotional Well-Being**	**Social Functioning**
Ratio MUFA/SFA	1.10 (0.16, 2.05) *	0.74 (0.21, 1.28) *	0.48 (0.01, 0.95) *	0.16 (−0.44, 0.77)
Alcohol	0.60 (−0.41, 1.62)	0.47 (−0.10, 1.04)	−0.14 (−0.63, 0.36)	0.05 (−0.60, 0.69)
Cereal	0.85 (−0.13, 1.83)	−0.39 (−0.95, 0.16)	0.14 (−0.34, 0.63)	−0.13 (−0.76, 0.49)
Vegetables	−0.42 (−1.37, 0.54)	0.66 (0.12, 1.20) *	0.24 (−0.23, 0.71)	−0.11 (0.72, 0.50) *
Fruits	0.98 (0.01, 1.95) *	1.67 (1.12, 2.21) *	1.22 (0.74, 1.70) *	−0.59 (−1.21, 0.03)
Fish	0.96 (0.02, 1.89) *	0.95 (0.42, 1.48) *	0.46 (−0.01, 0.92)	−0.29 (−0.89, 0.30)
Legumes	0.68 (−0.25, 1.60)	0.91 (0.39, 1.43) *	−0.10 (−0.56, 0.36)	0.10 (−0.49, 0.69)
Dairy	−0.79 (−1.78, 0.21)	−0.35 (−0.91, 0.21)	−0.63 (−1.12, 0.14)	0.13 (−0.50, 0.76)
Meat	−0.46 (−1.43, 0.52)	0.09 (−0.46, 0.65)	0.15 (0.33, 0.63) *	−0.25 (−0.87, 0.37)

Each β-coefficient translates to the associated SF-36 points gained or lost in the presence of each individual factor. Individual regression models were performed for each of the summary scales, these are adjusted for the variables in this table in addition to each item of the Mediterranean ,diet score and total energy (kcal/day). Abbreviations: BMI, Body mass index; METs, Metabolic equivalent of task. Prev, prevalence of the chronic disease. If no reference category is specified, it was therefore set to the absence of the condition. Statistically significant results ($p < 0.05$) are presented with "*" on the table.
